# Development of a Simple and Practical Screening Tool for Detection of Sarcopenia in Older People: The Bushehr Elderly Health Program

**DOI:** 10.3389/fmed.2021.655759

**Published:** 2021-04-13

**Authors:** Gita Shafiee, Afshin Ostovar, Saba Maleki Birjandi, Iraj Nabipour, Bagher Larijani, Ramin Heshmat

**Affiliations:** ^1^Chronic Diseases Research Center, Endocrinology and Metabolism Population Sciences Institute, Tehran University of Medical Sciences, Tehran, Iran; ^2^Osteoporosis Research Center, Institute of Endocrinology and Metabolism, Tehran University of Medical Sciences, Tehran, Iran; ^3^The Persian Gulf Marine Biotechnology Research Center, Bushehr University of Medical Sciences, Bushehr, Iran; ^4^Endocrinology and Metabolism Research Center, Endocrinology and Metabolism Clinical Sciences Institute, Tehran University of Medical Sciences, Tehran, Iran

**Keywords:** sarcopenia, screening tool, sensitivity, accuracy, iranian older population

## Abstract

**Background:** Sarcopenia is defined by the loss of muscle mass and function with a considerable prevalence which increases morbidity and mortality. We aimed to develop and validate a simple tool for screening of sarcopenia in Iranian older population.

**Methods:** In this study, we included 2,211 adults aged 60 years or older that participated in the stage II of Bushehr Elderly Health (BEH) program, a population-based prospective cohort study. We defined sarcopenia as reduced skeletal muscle strength plus low muscle mass. The study sample was divided into two parts; development set which were allocated to the development of the model (*n* = 1,499) and validation set (*n* = 712) were allocated to validation of the model.

**Results:** There were 22.9% of men and 23.2% women classified as having sarcopenia based on EWGSOP-2.After selection of variables, the final models named SarSA-Mod (Sarcopenia Scoring Assessment Models) were developed with area to under curves (AUC) of 0.82 (0.79–0.86) and 0.87 (0.84–0.90) in men and women, respectively. The final model included “age,” “weight,” and “calf circumference” in both sexes. The sensitivity and specificity and positive and negative predictive values for sarcopenia were 84.3, 76.0, 49.8, and 94.5% for women, 85.4, 64.8, 40.2, and 94.2% for men, respectively. The model performance was tested in the validation set with accuracy 91 and 84% among women and men, respectively.

**Conclusions:** Sarcopenia could be detected using SarSA-Mod, as a simple screening test with high accuracy among both sexes. Also, this screening test is valid, feasible, reliable and cost-effective compared to other tools.

## Introduction

Sarcopenia is a geriatric disease, characterized by loss of skeletal muscle mass and muscle function, leading to adverse effects such as physical disability, poor quality of life and increased mortality (1, 2). The range of the prevalence of sarcopenia is 5–13% among older people (3–5). In addition, with the increasing number of the aged people in the world, its prevalence will increase and it is often regarded as a global public health problem (6, 7).

Moreover, individuals with sarcopenia are not aware of the disease in the earlier stage but gradually, critical events in physical and functional disability occur (8). Therefore, early detection of individuals at risk of sarcopenia forms the basis for primary prevention in order to reduce the progress of sarcopenia and prevent its severe outcomes (8, 9).

European Working Group on Sarcopenia in Older People (EWGSOP) and Asian Working Group for Sarcopenia (AWGS) commonly recommended the use of diagnostic algorithms for sarcopenia and also, they recommended to use of dual-energy X-ray absorptiometry (DXA) and/or bioelectrical impedance analysis (BIA) (3) for diagnosing low muscle mass (1, 10). However, these tools and other methods such as magnetic resonance imaging (MRI) and computed tomography (CT) are not recommended as screening tools for the entire population. Besides, they are not available everywhere and need special training. Therefore, screening of all individuals according to EWGSOP or AWGS algorithms with DXA, CT and/or MRI are very costly, time-consuming, and impractical approaches for clinical practice in poor clinical settings (11).

Other screening tools were recently developed to identify older adults at higher risk for sarcopenia. The SARC-F questionnaire is a simple and easy for screening of sarcopenia in older adults (12). However, it has been validated in different population in the world, but the low sensitivity is a problem for a good screening tool. Therefore, for increasing of sensitivity some researchers added simple anthropometric parameters such as calf circumference to the SARC-F. Some studies showed that combination of calf circumference with this questionnaire can improve diagnostic accuracy of SARC-F (13). Another tool for screening of sarcopenia is known as the Mini Sarcopenia Risk Assessment (MSRA) (14) with high sensitivity and specificity compared to SARC-F (15).

Although, there are various screening tools for sarcopenia, there is no consensus on the best tool for all older people in the world and most of these methods have not tested with other ethnic populations. Therefore, in the present study, the aim was to develop a simple, cost-effective, non-invasive model of parameters to identify sarcopenia in order to facilitate sarcopenia screening in clinical setting of Iranian older population. Finally, the accuracy and diagnostic value of this model compared with other screening tools in a community-dwelling older adult population.

## Materials and Methods

### Study Design and Participants

The methodology of Bushehr Elderly Health (BEH) program has been previously described elsewhere (16). In summary, the BEH program is a prospective population-based cohort study aimed at determining the prevalence and risk factors of non-communicable diseases (NCD) among a representative sample of urban older population in Bushehr, South Iran. The target population of study was all people aged 60 years and over residing in the city of Bushehr. This population was about 10,000 persons according to District Health Center of Bushehr. We selected participants through a multi-stage, stratified cluster for BEH study. A total 3,000 people participated in the first Phase of this cohort. After 2.5 years, all participants were invited as the second stage of the BEH program for assessing of musculoskeletal disorders and cognitive impairment in these people (Supplementary Figure 1) (17). Until the time of the current study, 2,211 subject entered stage II. All participants signed a written informed consent and the Research Ethics Committee of Bushehr University of Medical Sciences approved the study.

### Measurement of Sarcopenic Parameters and Anthropometric Measurements

Body composition was measured using dual x-ray absorptiometry (DXA, Discovery WI, HologicInc, USA). Appendicular skeletal muscle mass (ASM) for each participant was derived as the sum of upper and lower limb muscle mass and the skeletal muscle mass index (SMI) as ASM/height^2^ (kg/m^2^).

Muscle strength was measured by handgrip strength, using a digital dynamometer. The participant seated, elbow at side and 90^0^ and the hand in a neutral position. The measurement was carried out three times for each hand and maximum grip strength was calculated by taking the highest measurement from both hands (18). Usual walking speed (m/s) on a 15 feet (4.57-meter) course was used as an objective measure of physical performance (1, 19). Heights and weights of participants were measured with a fixed stadiometer and a digital scale according to the standard protocol with shoes removed and the participants wearing light clothing. Body mass index (BMI) was calculated as weight (kg) divided by squared height (m^2^). Waist circumference (WC) was measured at a point midway between the iliac crest and the lowest rib in standing position and hip circumference was measured at the most part of the hip, using a flexible tape,. Upper arm circumference was measured at the midpoint between the olecranon process and the acromion of right arm, as well as, forearm circumference was measured from the widest level with the arm hanging freely at the side. Mid-thigh circumference was measured at a midpoint between trochanterion (top of the thigh bone, femur) and tibialelaterale (top of the tibia bone) of right thigh. Calf circumference was measured at the widest level while the participant was standing upright. All measurements were read to the nearest 0.1 cm.

Blood pressure (BP) was measured twice in a seated position after 15 min rest using a standard mercury sphygmomanometer. The average of the two measurements was considered as the participant's blood pressure.

### Definition of Sarcopenia

Sarcopenia was defined as low muscle strength plus reduced skeletal muscle mass based on the criteria set by EWGSOP-2 (2) which recommends the use of reference data to determine cut-off points for muscle mass, along with AWGS (10). In a recent study, reference data from a normative Iranian population are available for detecting sarcopenia. Based on these data, the cut-off values for low SMIs were 7.0 kg/m2 and 5.4 kg/m2 among men and women, respectively (20). The muscle strength were handgrip strength <26 kg for men and <18 kg for women; while the cut-off value for low physical performance was a usual walking speed <0.8 m/s for both genders (10, 21). Using these cut-off points, sarcopenic individuals were identified.

### Screening Tools

The SARC-F questionnaire and SARC-F with calf circumference were used to compare the new tool obtained from the current study. Strength, ambulation, rising from a chair, stair climbing and history of falling are five domains that are assessed. A score of four or more indicates a risk of sarcopenia (12). Another screening tool is SARCF-Calf that comprises five domains of the SARC-F and calf circumference. We used two cut-off points for calf circumference (CC) according to previous studies: (a) CC ≤31 cm for both genders and (b) CC ≤33 cm for women and CC ≤34 cm for men. The CC item is scored 0 points when it is above of the cut off and as 10 points if it is below or equals the cut points. A total score ≥11 indicates positive screening for sarcopenia (2, 21).

### Sarcopenia Scoring Assessment Models (SarSA-Mod)

In the current study, we developed a statistical model for screening sarcopenia. Proposed model: “Sarcopenia Scoring Assessment Model (SarSA-Mod)” is based on a prediction equation for screening sarcopenia regarding factors effect on this disease in our study population. SarSA-Mod has been built of three variables including age, weight and calf circumference in both genders.

Details on the methods of developing and validation of SarSA-Mod are explained in the statistical section.

### Statistical Analysis

Differences in between-group characteristics were examined by student's *t*-tests on the whole dataset.

To develop a statistical screening model to identify patients with sarcopenia, “True validation” or “holdout validation” method was used (22). Using random sampling, the study sample was divided into two parts; 67% of the cases (*n* = 1,499), called “development set,” which were allocated to the development of the model as in true validation, and 33%; one third of the dataset (*n* = 712), named “validation set” were allocated to validation of the model. This method is a cross validation as named the holdout model. After dividing the dataset into two sets as earlier mentioned, analysis for developing the model in the development set begun and all analysis were stratified by sex.

Candidate variables including age, waist circumference, hip circumference, thigh, upper arm circumference, calf circumference and also weight and BMI were selected based on previous studies, cost-effectiveness, feasibility and availability of variables to be measured, and the results of bivariate analysis.

Chi-square test was used to estimate the effect of each variable with the outcome (sarcopenia) as the dependent variable.

Logistic regression analysis was applied in the development of the final model. To choose the best model, we considered the goodness of fit of the models in both genders.

After selecting final model, ß coefficient of each variable was used to calculate its index weight. To discriminate the effect of each variable, the values were rounded to the nearest integer and multiplied by 10, and the final values were used to develop a suitable scoring model.

The ability of the model to separate those with sarcopenia from those without sarcopenia was evaluated using receiver operating characteristic (ROC) curves and the area under the ROC curve. A suitable cut-off point was selected the maximum value of Youden's index with regards to sensitivity and specificity for the model SarSA-Mod (23, 24).

Then, sensitivity, specificity, positive and negative predictive values (PPV & NPV) and the accuracy of the scores were evaluated using the “validation set,” which was left aside so far and not engaged in the model development process. In order to select and validate the final criteria for our scoring model, the model was applied to the validation set, using ROC analyses.

All analysis was performed using SPSS (version 16; SPSS Inc., Chicago, IL, USA) and STATA (Release 12. Statistical software. College Station, Texas: STATA Corp LP). *P*-value < 0.05 was defined as being statistically significant.

## Results

There were 22.9% of men and 23.2% women classified as having sarcopenia based on EWGSOP-2. The characteristics of the participants by sex and sarcopenia status are shown in Table 1. Participants with sarcopenia had significantly lower height, weight, BMI, waist and hip circumferences, calf and thigh circumferences, upper arm and forearm circumferences than those with non-sarcopenia in both sexes. Also, those with sarcopenia were older and had smaller handgrip strength, ASM, SMI and usual gait speed compared to those without sarcopenia in both sexes (all *P* < 0.001). There were no differences in DBP in men and SBP in both sexes irrespective of the presence of sarcopenia.

**Table 1:** Characteristics of study population.

	**Men**			**Women**		
	**No Sarcopenia (*n* = 831)**	**Sarcopenia (*n* = 247)**	***P*-value**	**No Sarcopenia (*n* = 852)**	**Sarcopenia (*n* = 258)**	***P*-value**
Age (years)	68.13 ± 5.13	74.32 ± 7.38	<0.001	68.17 ± 5.66	71.85 ± 6.95	<0.001
Height (cm)	166.81 ± 6.03	162.85 ± 6.36	<0.001	152.86 ± 5.98	150.67 ± 6.36	<0.001
Weight (Kg)	74.74 ± 11.90	64.00 ± 9.96	<0.001	70.19 ± 11.45	54.15 ± 8.65	<0.001
BMI (Kg/m^2^)	26.85 ± 3.95	24.11 ± 3.36	<0.001	30.05 ± 4.76	23.79 ± 3.13	<0.001
Grip Strength (Kg)	33.55 ± 7.00	20.74 ± 3.72	<0.001	18.75 ± 5.05	13.54 ± 3.11	<0.001
Waist Circumference (cm)	98.56 ± 10.88	92.40 ± 10.89	<0.001	103.48 ± 11.23	90.26 ± 10.36	<0.001
Hip circumference (cm)	100.55 ± 7.21	95.60 ± 7.25	<0.001	108.23 ± 10.18	96.11 ± 7.83	<0.001
Thigh circumference (cm)	50.76 ± 5.80	47.05 ± 5.09	<0.001	53.82 ± 6.74	46.75 ± 6.17	<0.001
Calf circumference (cm)	36.05 ± 3.43	33.20 ± 2.83	<0.001	36.67 ± 3.97	31.63 ± 2.92	<0.001
Upper arm circumference(cm)	30.13 ± 3.18	27.53 ± 2.77	<0.001	31.33 ± 3.51	26.95 ± 2.99	<0.001
Forearm circumference (cm)	26.85 ± 2.26	24.62 ± 1.96	<0.001	25.46 ± 2.13	22.67 ± 2.19	<0.001
SBP (mmHg)	140.06 ± 18.95	140.32 ± 21.09	0.851	139.87 ± 18.96	138.09 ± 19.58	0.190
DBP (mmHg)	82.74 ± 8.42	81.60 ± 9.09	0.067	81.52 ± 8.32	79.82 ± 7.89	0.004
Appendicular muscle mass(Kg)	19.36 ± 2.57	16.24 ± 1.88	<0.001	14.07 ± 1.91	11.14 ± 1.23	<0.001
SMI (Kg/m^2^)	6.96 ± 0.80	6.12 ± 0.57	<0.001	6.03 ± 0.77	4.90 ± 0.37	<0.001
Usual gait speed (m/s)	1.00 ± 0.29	0.81 ± 0.29	<0.001	0.77 ± 0.30	0.72 ± 0.37	<0.001

*Data are shown as mean ± standard deviation. BMI, body mass index; SBP, systolic blood pressure; DBP, diastolic blood pressure; SMI, skeletal muscle mass index*.

Table 2 shows the discriminatory performances of the models based on the number of variables. The following predictors were considered including: age, weight, and calf circumference for both sexes. This table presents ß coefficient, standard error and index weight of each variable in the final model in both genders. ß coefficients were rounded and multiplied by 10 to develop the final models. The formulas of final models were [(0.2 * age(years)) − (1.7 * calf circumference(cm)) − (weight(Kg) + 92.56)] in women and [(1.4 * age(years)) − (1.2 * calf circumference(cm)) − (0.5 * weight(Kg)) − 37.42] in men.

**Table 2:** The results of the multivariate analysis and the development scoring system in development set.

**Variable**	**B coefficient (95% CI)**	**Standard error**	***P*-value**	**Score**
**Men**				
Age	0.14 (0.11 to 0.17)	0.0167	<0.001	1.4
Weight	−0.05 (−0.08 to −0.02)	0.0155	0.002	−0.5
Calf circumference	−0.12 (−0.22 to −0.006)	0.0552	0.040	−1.2
Constant	−3.742 (−7.09 to −0.39)	1.7105	0.029	
Pseudo R^2^	0.34			
**Women**				
Age	0.02 (−0.01 to 0.06)	0.017	0.10	0.2
Weight	−0.10 (−0.14 to −0.07)	0.018	0.000	−1
Calf circumference	−0.17 (−0.27 to −0.08)	0.049	0.000	−1.7
Constant	9.256 (5.620 to 12.862)	1.840	0.000	
Pseudo R^2^	0.31			

*CI, confidence interval*.

The ROC (receiver operating characteristic) curves analyses were performed on the final scoring models for both genders. The full models had values of area under curve (AUC) as 0.82 (95%CI: 0.79–0.86) for men and 0.87 (95% CI: 0.84–0.90) for women. Based on the ROC curves analyses, cut-off points of −19.07 and −14.19 were selected for men and women, respectively; as appropriate for the models.

The score of −19.07 correctly classified 69.4% of men with sarcopenia with a sensitivity of 85.4% and a specificity of 64.8% and also, among women the score of −14.19 correctly classified 77.8% of women with a sensitivity of 84.3% and specificity of 76.0%.

Next, the models were internally validated using the validation set. The performance of the models did not differ significantly in the development and validation datasets. In the validation sample, the model for men had a sensitivity of 87.6% and specificity of 62.8%, and correctly classified 69.2% of cases; and the model for women had sensitivity of 89.1% and specificity of 77.7%, and correctly classified 80.7% of patients (Table 3).

**Table 3:** Performance of the SarSA-Mod in the development and validation samples.

**Samples**	**Area under Curve**	**Sensitivity (%)**	**Specificity (%)**	**Positive predictive value (%)**	**Negative predictive value (%)**	**Correctly classified (%)**
**Development set**						
Men	0.82 (0.79–0.86)	85.4 (79.0–90.5)	64.8 (60.7–68.7)	40.2 (34.9–45.6)	94.2 (91.4–96.3)	69.4
Women	0.87 (0.84–0.90)	84.3 (77.9–89.5)	76.0 (72.3–79.4)	49.8 (43.8–55.8)	94.5 (92.0–96.4)	77.8
**Validation set**						
Men	0.84 (0.80–0.89)	87.6 (79.0–93.7)	62.8 (56.6–68.7)	44.8 (37.3–52.5)	93.6 (88.9–96.8)	69.2
Women	0.91 (0.88–0.94)	89.1 (80.9–94.7)	77.7 (72.2–82.6)	58.2 (49.6–66.4)	95.4 (91.7–97.8)	80.7

*SarSA-Mod, Sarcopenia Scoring Assessment Model*.

Table 4 shows the comparison between the screening methods; SarSA-Mod, SARC-F, SARCF-Calf (31 cm) and SARCF-Calf (33/34 cm) in the total population. The current tool could identify 86% of men or women with sarcopenia, but the SARC-F questionnaire classified only 42% of men and 45% women as screening targets. However, when calf circumference added to SARC-F with both cut-off points, the tools can identify sarcopenic patients better than SARC-F alone. SarSA-Mod was superior to SARC-F and SARCF-Calf in terms of AUC, sensitivity and NPV.

**Table 4:** Comparison between the screening methods; SarSA-Mod, SARC-F, SARCF-Calf (31 cm), and SARCF-Calf (33/34 cm).

	**Area under Curve**	**Sensitivity (%)**	**Specificity (%)**	**Positive predictive value (%)**	**Negative predictive value (%)**
**SARC-F**					
Men	0.42 (0.37–0.47)	13.8 (9.8–18.8)	95.9 (94.3–97.1)	50.0 (37.6–62.4)	78.9 (76.2–81.4)
Women	0.45 (0.41–0.49)	29.6 (24.1–35.6)	73.4 (70.3–76.4)	25.3 (20.4–30.6)	77.6 (74.4–80.3)
Total	0.57 (0.54–0.60)	21.9 (18.3–25.7)	84.5 (82.7–86.2)	29.0 (25.2–34.8)	78.2 (76.3–80.1)
**SARCF-Calf (31 cm)**					
Men	0.49 (0.43–0.54)	9.4 (6.0–13.7)	98.7 (97.6–99.3)	67.7 (49.5–82.6)	78.5 (75.9–81.0)
Women	0.62 (0.57–0.66)	28.4 (23.0–34.3)	96.1 (94.6–97.3)	68.7 (59.1–77.5)	81.6 (79.0–83.9)
Total	0.64 (0.62–0.67)	19.1 (15.7–22.8)	97.4 (96.5–98.1)	68.6 (60.2–76.1)	80.0 (78.2–81.7)
**SARCF-Calf (33/34 cm)**					
Men	0.61 (0.56–0.66)	27.2 (21.8–33.3)	92.9 (90.9–94.5)	53.2 (44.1–62.1)	81.1 (78.4–83.5)
Women	0.73 (0.69–0.77)	48.6 (42.4–54.9)	89.6 (87.4–91.6)	58.7 (51.8–65.4)	85.2 (82.7–87.5)
Total	0.73 (0.70–0.76)	38.2 (33.9–42.6)	91.2 (89.8–92.5)	56.6 (51.2–62.0)	83.1 (81.3–84.8)
**SarSA-Mod**					
Men	0.83 (0.80–0.86)	86.2 (81.3–90.3)	64.3 (60.9–67.5)	41.8 (37.5–46.2)	94.0 (91.7–95.8)
Women	0.88 (0.86–0.90)	86.1 (81.2–90.0)	76.5 (73.5–79.3)	52.6 (47.7–57.5)	94.8 (92.8–96.3)
Total	0.86 (0.84–0.88)	86.1 (82.8–89.0)	70.5 (68.2–72.6)	46.7 (43.4–49.9)	94.4 (93.0–95.6)

*SarSA-Mod, Sarcopenia Scoring Assessment Model*.

**Figure 1:**
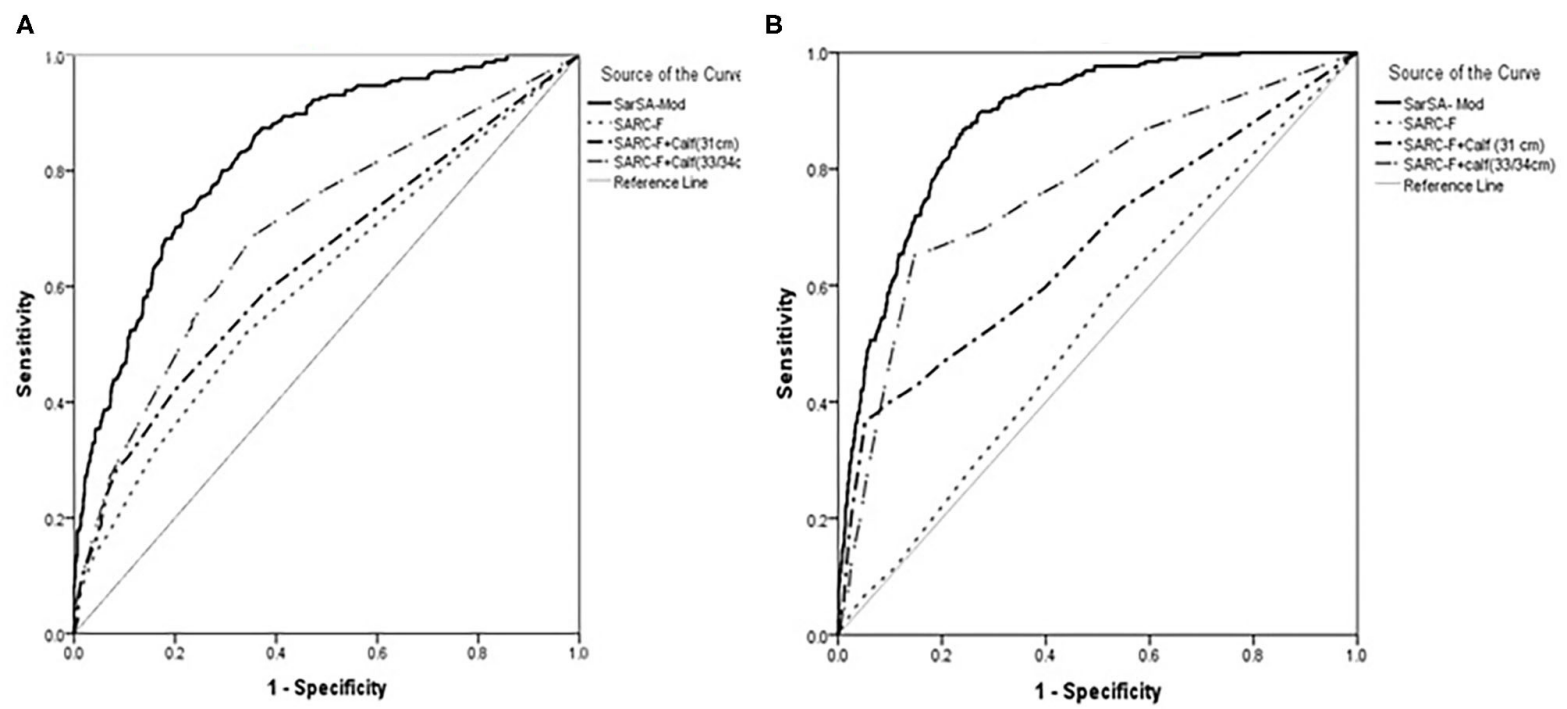
The receiver operating characteristic (ROC) curve for SarSA-Mod (Sarcopenia Scoring Assessment Model), SARC-F, SARCF-Calf (31 cm) and SARCF-Calf (33/34 cm) in the total population in **(A)** men and **(B)** women.

The AUC of SarSA-Mod, SARC-F, SARCF-Calf (31 cm) and SARCF-Calf (33/34 cm) in both sexes of total population are given in Figure 1. The AUCs of SarSA-Mod, SARC-F, SARCF-Calf (31 cm) and SARCF-Calf (33/34 cm) were 0.88, 0.53, 0.67 and 0.76 for women, 0.83, 0.61, 0.64 and 0.70 among men, respectively (*P* < 0.001).

Also we compared our models with calf circumference alone. The AUCs of calf circumference were 0.20, 0.15, and 0.25 for total population, women and men, respectively (Supplementary Table 1).

## Discussion

In the present study, the sarcopenia screening models for men and women were developed and validated in an Iranian population. Multivariate models were created based on selected variables and good discrimination ability of the models was found with the AUC of 0.82 and 0.87 for men and women, respectively. Based on the ROC curves analyses, the cut-off points of −19.07 and −14.19 were selected for men and women, respectively, and these scores could correctly classify sarcopenic patients with excellent discriminatory power.

To develop SarSA-Mod, important variables associated with sarcopenia or low muscle mass clinically or statistically significant, were examined. Of these factors, the best variables were selected as potential independent parameters of the models in both genders. First, a baseline model was identified according to age and weight which were important factors to develop the model in the previous studies (25, 26). Then, the incremental effect of anthropometric parameters to predict sarcopenia was investigated.

Among the anthropometric factors, measurement of calf circumference was simple and feasible and remained in our multivariate models in both genders. Studies have reported that calf circumference was highly correlated with muscle mass in both genders (27, 28). Also, generally, the extremities have a lower fat mass than other body sites (29). So, calf circumference can be used as a replacement indicator of muscle mass for diagnosing sarcopenia.

Therefore, SarSA-Mods were developed based on simple variables including age, weight, and calf circumference to the final model in both sexes. It seems that SarSA-Mods can be easily used in a primary care setting for a screening of sarcopenia in the general population.

In the present study, a scoring system was developed for screening sarcopenia using an index weight of each variable from linear regression analyses. However, several studies attempted for estimation of muscle mass by a variety of variables especially anthropometric parameters (30–32), but few studies developed models with varying degrees of accuracy for sarcopenia which was defined based on muscle mass with muscle function (12, 33).

The most common screening tool for sarcopenia is a five-domain questionnaire, called SARC-F (12). This tool is a simple and quick method and does not require complex measurements. Previous studies showed that the SARC-F could predict adverse outcomes such as hospitalization, poor quality of life, and death (34, 35). However, a major weakness of this tool is its low sensitivity which is confirmed by our results and other studies (13, 36). The low sensitivity of the SARC-F questionnaire limits its use as a screening tool for sarcopenia because it may miss diagnosing subjects who have sarcopenia (9). For this reason, a research group added calf circumference to the SARC-F to improve diagnostic accuracy and sensitivity of the original SARC-F (37). The findings of this study showed that SARCF-Calf had higher sensitivity and accuracy than SARC-F alone. Similar results from other studies were reported that the addition of calf circumference could increase sensitivity (13, 38). In contrast, a study reported that SARCF-Calf had no superiority for sensitivity but improved diagnostic accuracy and specificity (39). In our study, two different cut-off points (40, 41) used in the screening of sarcopenia; 31 cm for both genders, and 33 cm for women, and 34 cm for men. Our results indicate that although both SARCF-calf (31 cm) and SARCF-calf (33/34 cm) improve sensitivity and diagnostic accuracy of SARC-F, there are sensitivity levels of 19.1% −38.2% and accuracy levels of 0.64–0.73. In line with previous reports (13, 40), our findings showed that however, SARCF-Calf has better overall accuracy and sensitivity than SARC-F, but as a screening tool is not perfect.

Some studies exist that have developed the models incorporating the use of the anthropometric equation for muscle mass (30, 31). Although, this score has high accuracy for detecting of sarcopenia, these studies attempted to diagnose sarcopenia, according to the recent definitions of sarcopenia, they require the presence of low muscle mass as well as muscle function. So, the present study developed statistical models in both genders for the screening of sarcopenia, which was defined based on muscle mass and muscle function. Also, Ishii and et al. developed a rapid screening test including age, grip strength, and calf circumference for detecting sarcopenia in an Asian population (33). Although this model is very accurate for sarcopenia, the measurement of muscle strength in many medical centers is not feasible due to the lack of dynamometer. We used the variables in our equation can be measured easily and economically in the most clinics even with poor resource.

The discriminative performance of SarSA-Mod was significantly superior to that of SARC-F (AUC = 0.86 and 0.57, respectively, *P* < 0.001). Additionally, SarSA-Mod showed higher sensitivity and NPV than the SARC-F and SARCF-Calf in both genders and total subjects. Therefore, SarSA-Mod as a simple, non-invasive, and feasible tool, with high sensitivity and accuracy is better than SARC-F and SARCF-Calf for the detection of sarcopenia.

The present results have to be interpreted within the context of strengths and potential limitations. First, the studied population was sampled from an urban population; as a result, the study's findings might not be generalizable to the rural population. Second, our models were developed in a cross-sectional cohort and similarly validated in a study set on the same population. There is a need for establishing the external validity of the models in other study populations.

To the best of our knowledge, the current study is the first to develop and validate a sarcopenia screening model for Middle-East older people. Since SarSA-Mod is easy to calculate with simple variables, it is a useful screening model for sarcopenia in a primary care setting. The scores of SarSA-Mod can be used as an effective screening tool and help in identifying people with sarcopenia for interventions to prevent further adverse events.

## Data Availability Statement

The raw data supporting the conclusions of this article will be made available by the authors, without undue reservation.

## Ethics Statement

The studies involving human participants were reviewed and approved by Research Ethics Committee of Bushehr University of Medical Sciences. The patients/participants provided their written informed consent to participate in this study.

## Author Contributions

All authors listed have made a substantial, direct and intellectual contribution to the work, and approved it for publication.

## Conflict of Interest

The authors declare that the research was conducted in the absence of any commercial or financial relationships that could be construed as a potential conflict of interest.

## Supplementary Material

Supplementary Figure 1 The flowchart Bushehr Elderly Health (BEH) Program, phase I.

Supplementary Figure 2 The distributions and histograms of variables of SarSA-Mod in both genders.

Supplementary Table 1 The area under curve and other characteristics of calf circumference as screening tool for sarcopenia in the whole study population.
