# Supplementary figures and images for: Development of a Simple and Practical Screening Tool for Detection of Sarcopenia in Older People: The Bushehr Elderly Health Program
Source: Front Med (Lausanne). 2021 Apr 13;8:655759. doi: 10.3389/fmed.2021.655759 (PMC8076573; doi:10.3389/fmed.2021.655759)

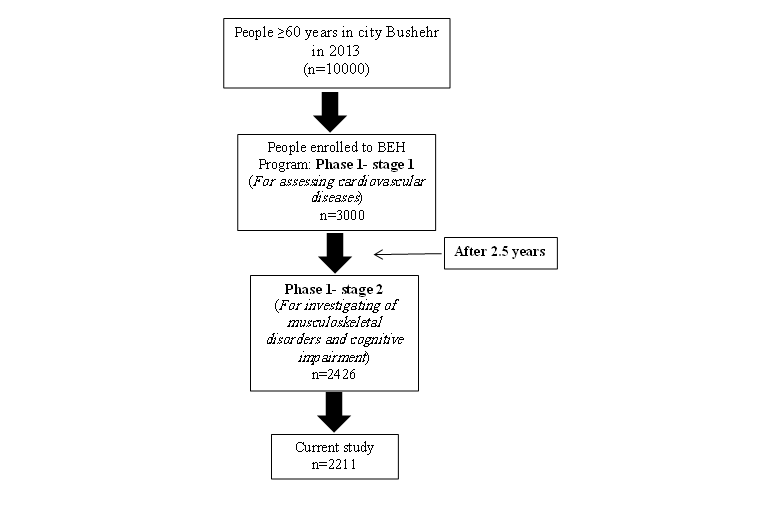

Supplement: Supplementary Figure 1 — The flowchart Bushehr Elderly Health (BEH) Program, phase I. [file Image_1.PNG]

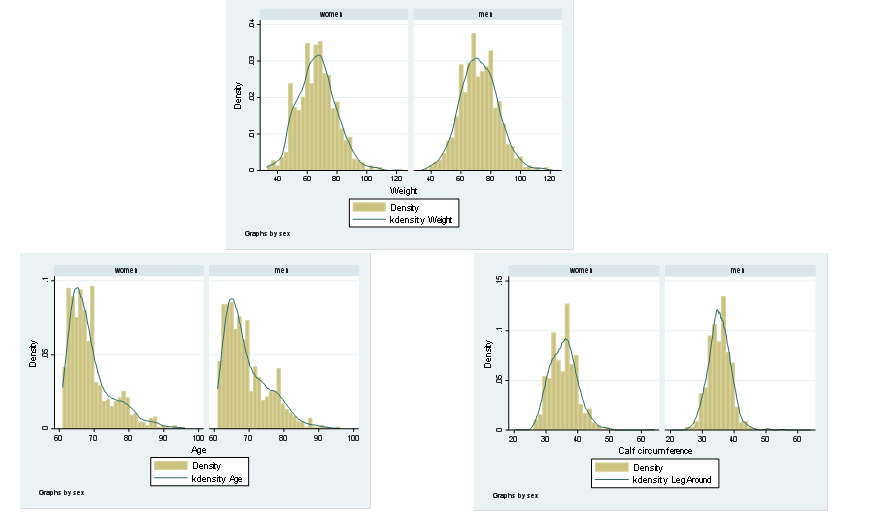

Supplement: Supplementary Figure 2 — The distributions and histograms of variables of SarSA-Mod in both genders. [file Image_2.PNG]
