# Supplementary material for: Development of a Simple and Practical Screening Tool for Detection of Sarcopenia in Older People: The Bushehr Elderly Health Program
Source: Front Med (Lausanne). 2021 Apr 13;8:655759. doi: 10.3389/fmed.2021.655759 (PMC8076573; doi:10.3389/fmed.2021.655759)
Supplement: Supplementary Table 1 — The area under curve and other characteristics of calf circumference as screening tool for sarcopenia in the whole study population. [file Table_1.docx]

| **Supplementary Table 1. The area under curve and other characteristics of calf circumference as screening tool for sarcopenia in the whole study population** | | | | | |
| --- | --- | --- | --- | --- | --- |
|  | **Area under Curve** | **Sensitivity (%)** | **Specificity (%)** | **Positive predictive value (%)** | **Negative predictive value (%)** |
| **Calf circumference** |  |  |  |  |  |
| **Men** | 0.25(0.22-0.28) | 16.2(11.8-21.3) | 95.1(93.5-96.5) | 50.0(38.6-61.3) | 79.2(76.6-81.7) |
| **Women** | 0.15(0.12-0.17) | 36.8(30.9-43.2) | 94.9(92.2-96.3) | 68.8(60.4-76.4) | 83.2(80.7-85.5) |
| **Total** | 0.20(0.17-0.22) | 26.7(22.9-30.8) | 95.0(93.9-96.0) | 61.9(55.1-68.4) | 81.2(79.4-82.9) |
